# Pirfenidone suppresses polarization to M2 phenotype macrophages and the fibrogenic activity of rat lung fibroblasts

**DOI:** 10.3164/jcbn.17-111

**Published:** 2018-04-11

**Authors:** Michihito Toda, Shinjiro Mizuguchi, Yukiko Minamiyama, Hiroko Yamamoto-Oka, Takanori Aota, Shoji Kubo, Noritoshi Nishiyama, Toshihiko Shibata, Shigekazu Takemura

**Affiliations:** 1 Department of Thoracic Surgery, Osaka City University Hospital, 1-4-3 Asahimachi, Abeno-ku, Osaka 545-8585, Japan

**Keywords:** pirfenidone, anti-fibrosis, M2 macrophage

## Abstract

Pirfenidone is a representative medication to treat interstitial pulmonary fibrosis. Researchers reported pirfenidone (>100 µg/ml) significantly suppressed fibroblast growth *in vitro*. However, clinically, the maximum concentration of pirfenidone in the blood is approximately 10 µg/ml. We hypothesized there might be an additional mechanism of pirfenidone to fibroblasts indirectly. Macrophages are known to control the activation of fibroblasts via the regulation of inflammatory M1 and suppressive M2 polarization. The aim of this study was to investigate the effects of pirfenidone on alveolar macrophage polarization. Rat alveolar macrophages (NR8383) were stimulated *in vitro* with lipopolysaccharide (LPS) + interferon (IFN)-γ, or interleukin (IL)-4 + IL-13. Expression of M1 and M2 markers and supernatant’s levels of TGF-β1 were assessed after pirfenidone treatment (0–100 µg/ml). Treatment with LPS + INF-γ or IL-4 + IL-13 significantly increased the expression of M1 and M2 markers, respectively. In macrophage polarization assays, pirfenidone significantly reduced the expression of M2 markers at concentrations greater than 10 µg/ml but had no effect on the expression of M1 markers. At these concentrations, pirfenidone significantly reduced TGF-β1 levels in NR8383 culture supernatants. In rat lung fibroblasts treated with NR8383 culture supernatants, pirfenidone significantly suppressed proliferation, and the collagen mRNA and protein levels. In conclusion, our results demonstrated that pirfenidone suppressed polarization to M2 macrophages at clinically relevant concentrations and suppressed the rat lung fibroblasts fibrogenic activity.

## Introduction

Idiopathic pulmonary fibrosis (IPF) is a chronic, progressive and inevitably fatal scarring lung disease with a poor prognosis.^(1)^ The etiology and the pathogenesis of IPF are still incompletely understood. The representative therapeutic drugs approved for patients with IPF are Pirfenidone (PFD) and Nintedanib.^(2)^ The anti-fibrotic properties of Nintedanib, an intracellular tyrosine kinase inhibitor, have been attributed to its inhibitory effects on the platelet derived growth factor, fibroblast growth factor and vascular endothelial growth factor receptors.^(3)^ PFD is an orally active small molecule comprising a modified phenyl pyridine that has been demonstrated clinically to reduce the mortality rate in IPF patients.^(4)^ PFD exhibits well-documented anti-fibrotic and anti-inflammatory activities in a variety of animal and cell-based models.^(5–7)^ However, the mechanism of action and molecular targets of PFD remain poorly understood.^(8)^

Some clinical reports have shown that oral administration of PFD significantly improves survival rate and respiratory function in IPF patients.^(9,10)^ The recommended oral dose of PFD is 200–600 mg, three times a day. The maximum plasma concentration following an oral dose of 200 mg PFD has been reported to be 3.88 ± 0.82 µg/ml and with a dose of 600 mg PFD, 10.57 ± 1.78 µg/ml.^(11)^ From these data, the maximum plasma concentration of PFD in the human body can be calculated to be approximately 3–13 µg/ml. Although several studies have reported that PFD can suppress fibroblast growth directly *in vitro*, they required PFD concentrations of more than 100 µg/ml, which greatly exceeds maximal plasma concentrations expected for this drug in the clinical setting.^(12,13)^ Moreover, low concentrations of PFD (less than 10 µg/ml) have not been reported to be effective in inhibiting fibroblast growth in *in vitro* studies.^(12)^ Given this discrepancy in the efficacy of PFD between the *in vivo* and *in vitro* setting, we hypothesized that there might be an additional mechanism of PFD action besides its direct inhibitory effect on fibroblasts.

Alveolar macrophages play a key role in the progression of pulmonary fibrosis.^(14)^ Macrophages comprise a heterogeneous population of cells with diverse functions and phenotypic plasticity. However, they can broadly be classified as belonging to either the M1 (classically activated) phenotype or the M2 (alternatively activated) phenotype.^(15)^ M1 macrophages are known to predominate during the progression of the inflammatory response.^(14)^ They release pro-inflammatory chemokines that exacerbate the injury, amplify the inflammatory response, and contribute to fibroblast proliferation and the recruitment of fibrocytes.^(16)^ Following the acute phase of inflammation, Th2 cytokines [e.g., interleikin-4 (IL-4) and interleikin-13 (IL-13)] are produced to promote the polarization and recruitment of M2 macrophages.^(17)^ Furthermore, M1 macrophages recognize and phagocytose apoptotic cells, and also promote macrophage alternative activation at the site of inflammation.^(18)^ In contrast to M1 macrophages, M2 macrophages induced by Th2 cytokines are intended to create an anti-inflammatory environment and promote healing and wound regeneration. However, when the lesion is persistent, M2 macrophages adopt an important pro-fibrotic role and these cell populations are known to secrete large amounts of pro-fibrotic factors such as transforming growth factor-β (TGF-β).^(19)^ From these insights, we sought to determine whether PFD suppressed inflammation by suppressing macrophage polarization towards the M1 phenotype, and whether PFD had an indirect inhibitory effect on fibroblast proliferation by suppressing macrophage polarization towards the M2 phenotype.

## Methods

### Reagents

PFD was kindly provided by Shionogi & Co., Ltd. (Osaka, Japan). Ham’s F-12K was purchased from Wako (Osaka, Japan), Dulbecco’s modified Eagle’s medium (DMEM) from Sigma-Aldrich (St. Louis, MO), fetal bovine serum (FBS) from Equitech-Bio, Inc. (Kerrville, TX), and penicillin and streptomycin from Gibco Invitrogen (NY). The protease inhibitor cocktail was from Roche Diagnostics (Mannheim, Germany), the Cell Counting Kit-8 from Dojindo (Kumamoto, Japan), and the BCA protein assay kits from Wako (cat. #PDG6489). Lipopolysaccharide (LPS) (E coli serotype 055: B5) was from Difco Laboratories (Detroit, MI), recombinant rat interferon-γ (IFN-γ) from Itsi Biosciences (Johnstown, PA), and recombinant rat IL-4 and recombinant rat IL-13 from Peprotech (Rocky Hill, NJ). Block ACE blocking reagent was from DS Pharma Biomedical Co., Ltd. (Osaka, Japan). The anti-inducible nitric oxide synthase (iNOS) mouse monoclonal antibody was from BD Bioscience (Franklin Lakes, NJ; cat. #610328), and the anti-TNF-α rabbit polyclonal antibody from Peprotech (cat. #500-P72). The anti-mannose receptor (CD206) rabbit polyclonal antibody (cat. #ab64693) and the anti-chitinase 3 like protein 3 + chitinase 3 like protein 4 (YM-1) rabbit polyclonal antibody (cat. #ab192029) were from Abcam (Cambridge, UK). The anti-transferrin receptor rabbit polyclonal antibody was from Bioss antibodies (Woburn, MA; cat. #AD082519), the anti-iron regulatory protein-1 (IRP-1) rabbit polyclonal antibody from Bioworld Technology (Saint Louis Park, MN; cat. #XCJ44121), and polyclonal secondary antibodies were from Dako (Glostrup, Denmark). The anti-collagen type 1 rabbit polyclonal antibody was from Rockland Immunochemicals, Inc. (Limerick, PA; cat. #38928). The anti-heat shock protein 47 (HSP47) mouse monoclonal antibody was from ENZO Life Science, Inc. (Farmingdale, NY; cat. #03031509). The enhanced chemiluminescence immunoblot assay kit (ECL prime) was from GE Healthcare (Little Chalfont, UK), and the enzyme-linked immunosorbent assay (ELISA) kit was from R&D Systems (Minneapolis, MN). The Nucleo Spin RNA kit was from Machery-Nagel (Dueren, Germany), and the ReverTra Ace qPCR RT Kit from Toyobo (Osaka, Japan). The TaqMan Universal PCR Master Mix, Optical reaction plates with adhesive covers, and the ABI Prism system were all from Applied Biosystems (Foster City, CA).

### Cells culture

The NR8383 rat alveolar macrophage cell line was purchased from the American Type Culture collection (Manassas, VA). Rat lung fibroblast (RLF) cell line was purchased from Sigma-Aldrich (St. Louis, MO). NR8383 cells were cultured in Ham’s F-12K medium supplemented with 15% FBS and RLF were cultured in DMEM with 10% FBS. All cells cultures were maintained at 37°C in a humidified atmosphere containing 5% CO_2_.^(20,21)^ The medium was changed every 3 days until the culture had reached 90% confluency. For experiments, cells were suspended in culture medium at a density of 1 × 10^6^ cells/ml. Passage 3–5 cells were used for all experiments. PFD was dissolved in distilled water containing 0.5% dimethyl sulfoxide (DMSO) and used at a final concentration of 0.1, 1, 10, 100 or 1,000 µg/ml.

### Proliferation assay

To determine the cytotoxic effects of PFD on NR8383 cells and RLFs, a viability assays was performed that measures the conversion of 2-(2-methoxy-4-nitrophenyl)-3-(4-nitrophenyl)-5-(2,4-disulfophenyl)-2H-tetrazolium, monosodium salt (WST-8) to a water-soluble orange formazan dye. Briefly, 5 × 10^3^ cells/well were seeded into a 96-well microtiter plate and incubated at 37°C in a 5% CO_2_, humidified atmosphere overnight. After 24 h, various dilutions of PFD (0, 10, 100 or 1,000 µg/ml) were added to the wells. NR8383 cells were then cultured for an additional 72 h and RLF for an additional 48 h in the presence of the drug before assessment of cell viability.

The WST-8 assay was also used to assess the proliferation of RLFs cultured in supernatants from NR8383 cells treated with PFD. RLFs (5 × 10^3^ cells/well) were seeded into a 96-well microtiter plate and cultured overnight. After 24 h, cell-free medium or conditioned medium from NR8383 cells treated with PFD (0, 1, 10 or 100 µg/ml) were added to the wells at a concentration of 10% (vol./vol.). The cells were incubated for 24 h and then Cell Counting Kit-8 reagent (10 µl) added to each well and the cells then incubated for an additional 2–3 h. The absorbance was measured at 450 nm using a Spectra Max 190 microplate reader (Molecular Devices, CA). All experiments were carried out in duplicate.

### Macrophage polarization under PFD treatment

 NR8383 cells grown in 12-well culture plates were cultured in Ham’s F-12K medium/15% FBS supplemented with PFD (0, 0.1, 1, 10 or 100 µg/ml) for 24 h before treatment with chemokines to promote macrophage polarization. After the 24 h PFD incubation, NR8383 cells were stimulated with LPS (5 ng/ml) + IFN-γ (10 ng/ml) or IL-4 (10 ng/ml) + IL-13 (10 ng/ml), to promote M1 and M2 polarization, respectively. After an additional 24 h, cell supernatants were collected for ELISA analysis and the cells washed twice with PBS and harvested in sample buffer (50 mM HEPES pH 7.5, 200 mM NaCl, 1 mM EDTA 2Na, 2.5 mM EGTA, 0.1% Tween-20, 10% glycerol, 0.1 mM Sodium orthovanadate, 1 mM Sodium fluoride, protease inhibitor cocktail) for subsequent analysis by western blotting.

### Western blotting

The protein concentration of samples was assessed by BCA protein assay. Equal amounts of protein (5 µg) were subjected to 10% SDS-PAGE and subsequently transferred onto polyvinylidene difluoride (PVDF) microporous membranes (Merck Millipore, Darmstadst, Germany) as described previously.^(18)^ Membranes were blocked for 30 min with 4% Block ACE, and incubated overnight at 4°C with antibodies directed against either iNOS and TNF-α (all at 1:1,000) as M1 phenotype markers,^(22)^ or CD206, transferrin receptor, YM-1 and IRP-1 (all at 1:1,000) as M2 phenotype markers,^(23–25)^ or collagen type 1 and HSP47 (all at 1:1,000) as collagen synthesis markers.^(6,26)^ The membranes were then washed in tris-buffered saline/0.5% Tween-20 (TBST) and incubated with appropriate alkaline phosphatase-conjugated secondary antibodies (1:20,000) for 1 h. Protein bands were detected using the ECL prime kit and the LAS-3000 chemiluminescence detection/luminescent image analyzer system (Fujifilm, Tokyo, Japan). Densitometric analysis of protein bands was performed using Image-J software.

### ELISA

TGF-β1 levels in culture supernatants obtained from NR8383 cells that had been pretreated with PFD (0, 0.1, 1, 10 or 100 µg/ml) before the induction of macrophage polarization were examined by ELISA. The optical density of each sample was measured at 450 nm using a Spectra Max 190 microplate reader. The background TGF-β1 level (480 pg/ml) for cell-free medium was subtracted from each experimental value to calculate the level of TGF-β1 produced by the NR8383 cells.

### Quantitative real-time PCR

Total RNA from RLFs cultured in either cell-free medium or conditioned media from NR8383 cells treated as described above (see ‘Macrophage polarization under PFD treatment’) was extracted using the Nucleo Spin RNA kit. cDNA was synthesized using the ReverTra Ace qPCR RT Kit. Gene-specific primers were used to examine the expression of *Col1a1* (Rn01463848_m1) and *HSP47* (Rn00567777_m1) mRNA by PCR using the Taq Man Universal PCR Master Mix. Reaction volumes of 20 µl were loaded into 96-well optical reaction plates with adhesive covers and reactions were performed using the ABI PRISM 7500 real-time PCR system (Applied Biosystems, CA). Glyceraldehyde-3-phosphate dehydrogenase (GAPDH, Rn99999916_s1) served as the control target gene for reaction efficiency. Results were analyzed using the comparative cycle threshold (ΔΔCt) method. The primers for these genes were from Thermo Fisher Scientific (Yokohama, Japan).

### Western blot analysis of collagen type 1 and HSP47 in RLFs

RLFs were cultured in DMEM with 10% FBS supplemented with cell-free medium or conditioned medium from NR8383 cells treated with PFD (0, 1, 10 or 100 µg/ml). After 48 h, RLF supernatants were collected, and the RLFs were washed twice with PBS and harvested in sample buffer as described above for subsequent analysis by western blotting.

### Statistical analysis

All values are presented as mean ± SD. Statistical analysis was performed using one-way ANOVA. *P* values <0.05 were considered to be statistically significant. Statistical analysis was performed with the JMP11.0 software package (SAS Institute Inc., Cary, NC).

## Results

### Effects of PFD on the viability of alveolar macrophages and lung fibroblasts

**Fig. 1:**
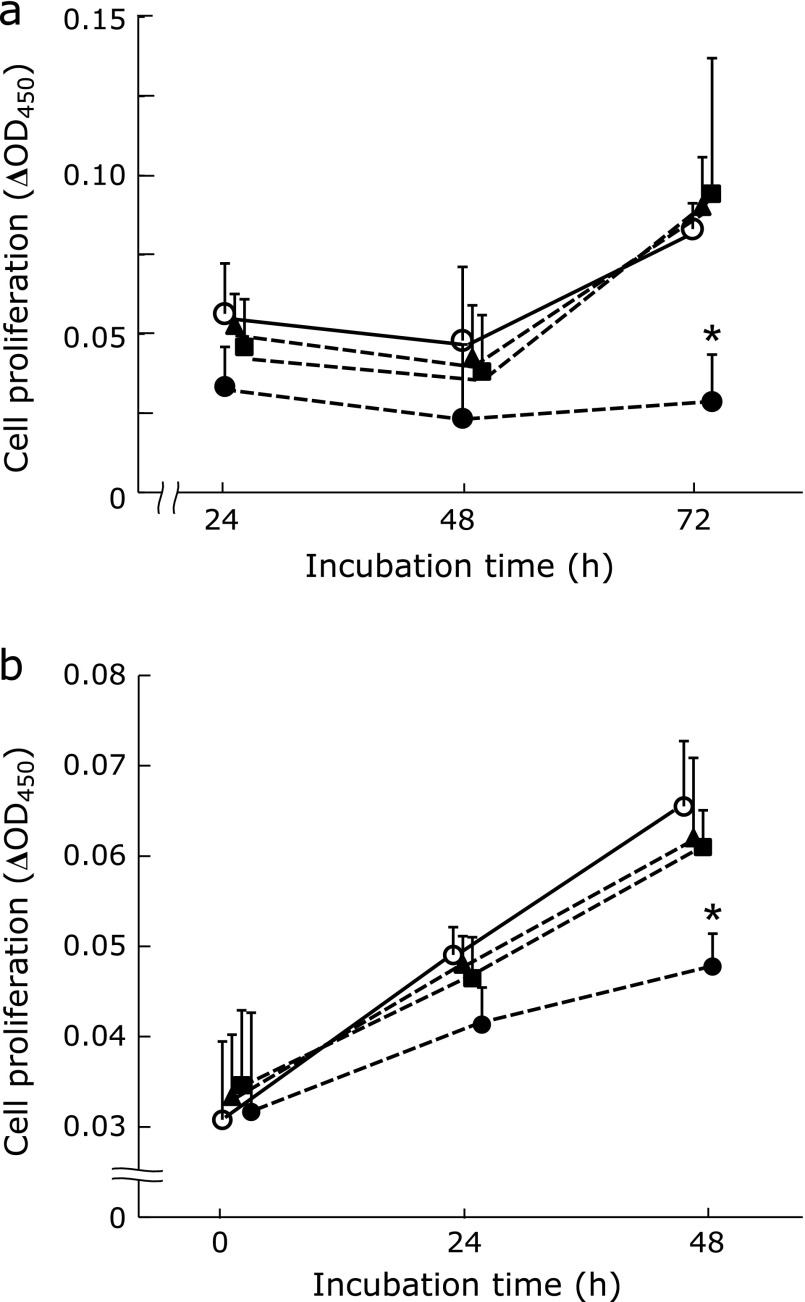
Cytotoxic effects of PFD on NR8383 cells and RLFs. Cell viability assays were performed to assess the cytotoxic effect of PFD on NR8383 cell and RLF proliferation. Plots show the effects of different concentrations of PFD on NR8383 cell (a) and RLF (b) growth over the indicated durations. Drug concentrations were as follows: ◯, Vehicle; ▲, PFD: 10 µg/ml; ■, PFD: 100 µg/ml; ●, PFD: 1,000 µg/ml. Experiments were carried out in duplicate. Data are means ± SD from six experiments. *p<0.05, when compared with vehicle.

The proliferative rate of NR8383 cells significantly decreased following treatment with PFD at a concentration of 1,000 µg/ml (Fig. 1a). However, PFD concentrations of 100 µg/ml or less had no effect on NR8383 cell proliferation. Therefore, subsequent experiments were performed using PFD concentrations of 100 µg/ml or less. In the non-stimulated (vehicle) NR8383 group, the OD value had increased 2.1 fold by 72 h. The proliferative rate of RLFs significantly decreased following treatment with 1,000 µg/ml PFD (55% of the OD value of the vehicle group; Fig. 1b). PFD concentrations of 100 µg/ml or less had no direct effects on the proliferation of RLFs.

### PFD has no effect on the polarization of alveolar macrophages towards the M1 phenotype

**Fig. 2:**
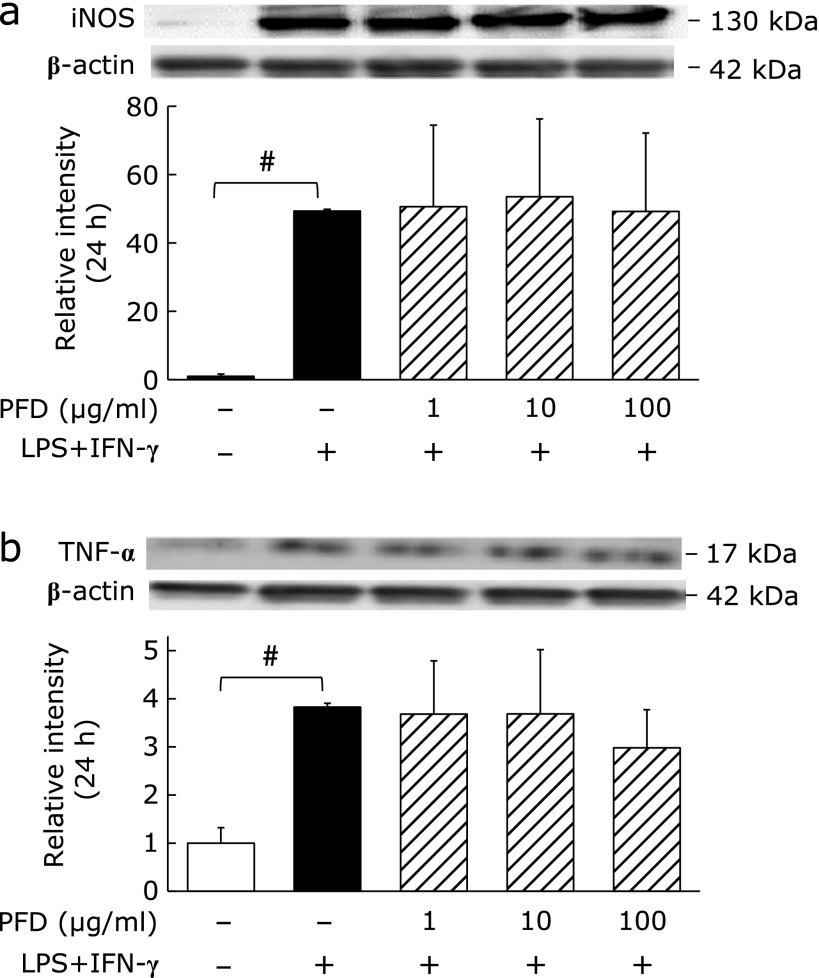
Effects of PFD on NR8383 cell polarization towards the M1 phenotype. Western blots and associated densitometry analysis of the expression of the M1 phenotypic markers (a) iNOS and (b) TNF-α in NR8383 cells pre-treated with the indicate concentrations of PFD for 24 h followed by stimulation with or without LPS (5 ng/ml) + IFN-γ (10 ng/ml) for an additional 24 h. Data are means ± SD from at least five experiments. ^#^*p*<0.01, when compared with the untreated group.

Expression of the M1 macrophage phenotypic markers iNOS and TNF-α significantly increased following treatment of NR8383 cells with LPS + IFN-γ (Fig. 2a and b). PFD treatment had no effect on the protein levels of these M1 phenotypic markers.

### PFD attenuates the expression of M2 phenotypic markers in alveolar macrophages

**Fig. 3:**
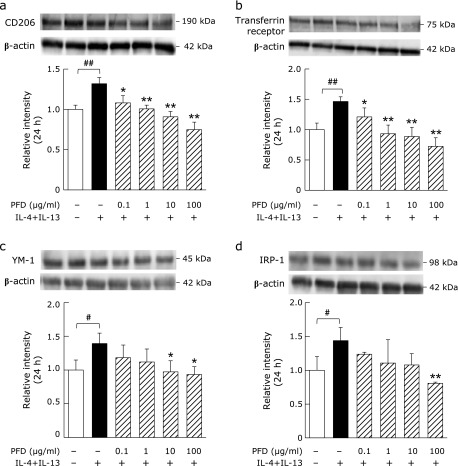
Effects of PFD on NR8383 cell polarization towards the M2 phenotype. Western blots and associated densitometry analysis of the expression of the M2 phenotypic markers (a) CD206, (b) transferrin receptor, (c) YM-1, and (d) IRP-1 in NR8383 cells pre-treated with the indicate concentrations of PFD for 24 h followed by stimulation with or without IL-4 (10 ng/ml) + IL-13 (10 ng/ml) for an additional 24 h. Data are means ± SD from at least five experiments. ^#^*p*<0.05 and ^##^*p*<0.01, when compared with the untreated group. *p<0.05 and **p<0.01, when compared with the IL-4 + IL-13 stimulated group.

Expression of the M2 macrophage phenotypic markers CD206, transferrin receptor, YM-1 and IRP-1 significantly increased after treatment of NR8383 cells with IL-4 + IL-13 (Fig. 3). PFD treatment significantly decreased the expression of these M2 phenotypic markers in a dose-dependent manner.

### PFD suppresses TGF-β1 release from M2 alveolar macrophages

**Fig. 4:**
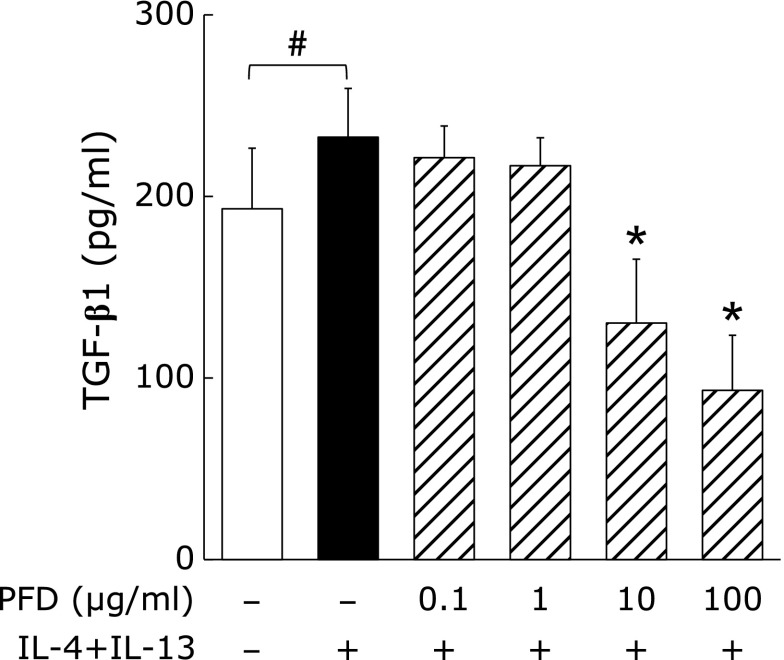
Effects of PFD on TGF-β1 levels in the culture supernatants of NR8383 cells stimulated with IL-4 + IL-13. NR8383 cells were pre-treated with the indicated concentrations of PFD for 24 h and then treated with or without IL-4 (10 ng/ml) + IL-13 (10 ng/ml) for an additional 24 h as indicated. TGF-β1 levels in culture supernatants were quantified by ELISA. Data are means ± SD from six experiments. ^#^*p*<0.05, when compared with the untreated group. *p<0.01, when compared with the IL-4 + IL-13 stimulated group.

TGF-β1 levels in the culture supernatants of NR8383 cells were significantly increased by IL-4 + IL-13. PFD dose-dependently attenuated this increase in TGF-β1 at concentrations of 10 µg/ml or more (Fig. 4). Moreover, TGF-β1 levels in the 10 µg/ml and 100 µg/ml PFD treatment groups decreased to 70% and 50% of that of the naïve cell group, respectively.

### PFD attenuates the proliferative effects of M2 alveolar macrophage culture supernatants on lung fibroblasts

**Fig. 5:**
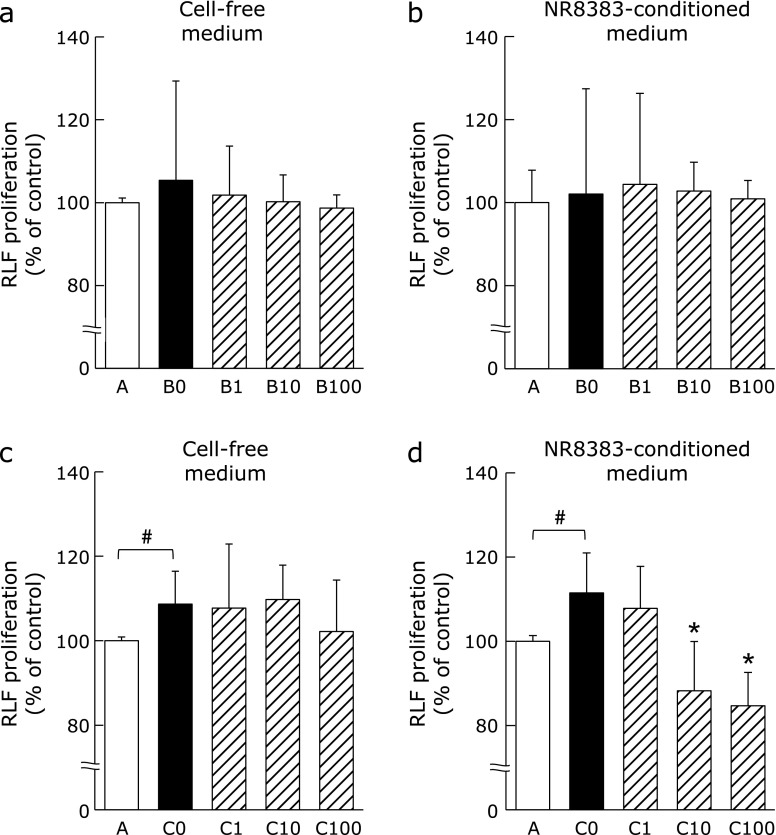
Effects of conditioned media from PFD-treated NR8383 cells on RLF proliferation. RLF proliferation following 24 h culture in either (a) 10% cell-free medium containing LPS (5 ng/ml) + IFN-γ (10 ng/ml) and PFD (0, 1, 10 or 100 µg/ml), (b) 10% conditioned medium from NR8383 cells stimulated with LPS + IFN-γ after 24 h pre-treatment with PFD, (c) 10% cell-free medium containing IL-4 (10 ng/ml) + IL-13 (10 ng/ml) and PFD or (d) 10% conditioned medium from NR8383 cells stimulated with IL-4 + IL-13 after 24 h pre-treatment with PFD. Treatments are as follows; A: 10% cell-free medium or conditioned medium with no stimulation; B0, B1, B10, B100: 10% cell-free media or conditioned media containing LPS + IFN-γ and PFD (0, 1, 10 or 100 µg/ml); C0, C1, C10, C100: 10% cell-free media or conditioned media containing IL-4 + IL-13 and PFD (0, 1, 10 or 100 µg/ml). Data are means ± SD from six experiments. All experiments were conducted in duplicate. ^#^*p*<0.05, when compared with A group, and *p<0.05, when compared with C0 group.

RLF proliferation was unaffected by treatment with either 10% cell-free medium supplemented with LPS + IFN-γ and varying concentrations of PFD (Fig. 5a), or 10% conditioned medium obtained from NR8383 cell cultures stimulated with LPS + IFN-γ and varying concentrations of PFD (Fig. 5b). RLF proliferation significantly increased after stimulation with 10% cell-free medium containing IL-4 + IL-13, while 10% cell-free medium containing varying concentrations of PFD had no effect on proliferation (Fig. 5c). However, the proliferation of RLFs was significantly attenuated by 10% NR8383 cell conditioned media containing IL-4 + IL-13 and PFD at concentrations of 10 µg/ml or more (Fig. 5d).

### Effects of PFD on *Col1a1* and *HSP47* mRNA expression in lung fibroblasts

**Fig. 6:**
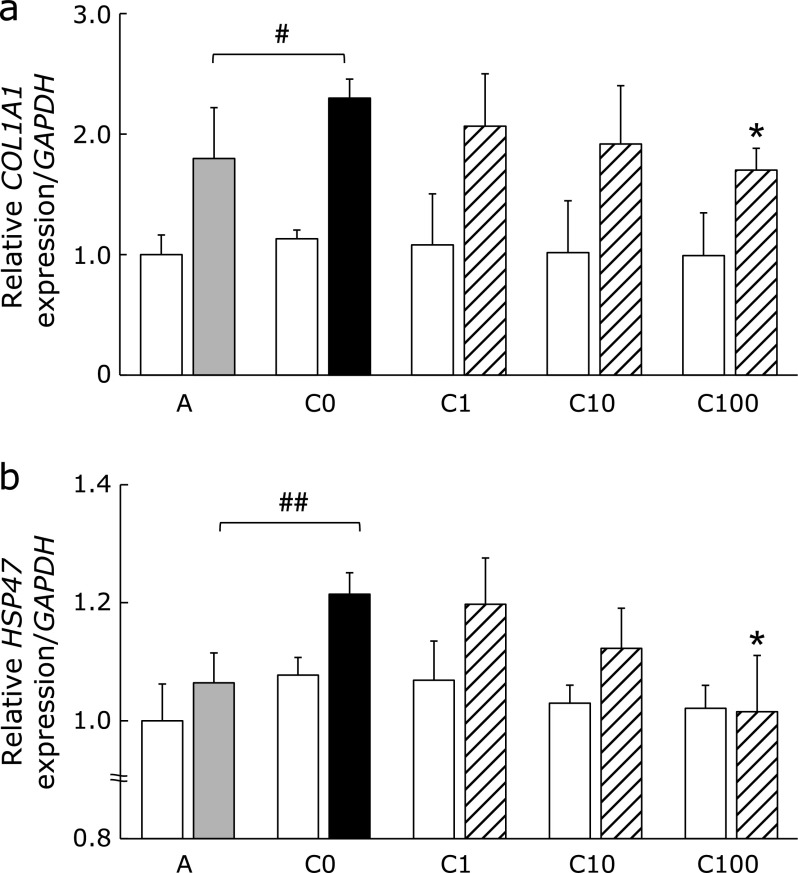
Effects of conditioned medium from PFD-treated NR8383 cells on *Col1a1* and *HSP47* mRNA expression in RLFs. RLFs were incubated at 37°C for 24 h. RLFs were stimulated with either 10% cell-free medium or conditioned media from NR8383 cells pre-treated with the indicated concentrations of PFD. *Col1a1* mRNA (a) and *HSP47* mRNA (b) expression were measured by real-time PCR and values were normalized to GAPDH. Treatments are as described Fig. 5. Each bars represents the following; open bar: cell-free medium; shaded bar: conditioned medium from naïve cells; closed bar: conditioned medium from NR8383 cells stimulated with IL-4 + IL-13; hatched bars: conditioned medium from NR8383 cells stimulated with IL-4 + IL-13 and indicated concentrations of PFD. Data are means ± SD from six experiments. ^#^*p*<0.05 and ^##^*p*<0.01, when compared with A group and *p<0.01, when compared with C0 group.

PFD treatment had no effect on *Col1a1* (Fig. 6a) and *HSP47* (Fig. 6b) mRNA expression in RLFs treated with 10% cell-free medium containing IL-4 + IL-13. However, *Col1a1* and *HSP47* RLF mRNA levels increased significantly following treatment with 10% conditioned medium from NR8383 cells stimulated with IL-4 + IL-13. The expression of these mRNAs was only significantly suppressed when RLFs were cultured with conditioned medium from NR8383 cells treated with the highest concentration of PFD (100 µg/ml).

### PFD attenuates the levels of collagen type 1 and HSP47 in lung fibroblasts

**Fig. 7:**
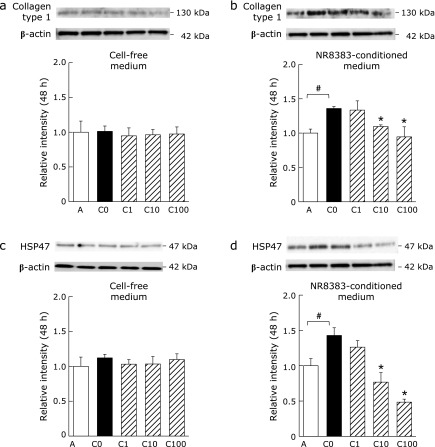
Effects of conditioned medium from PFD-treated NR8383 cells on collagen type 1 and HSP47 protein levels in RLFs. RLFs were treated as described in Fig. 6. The cell lysates were collected after 48 h of culture and examined by western blotting. (a) Collagen type 1 and (c) HSP47 after treatment with 10% cell-free medium. (b) Collagen type 1 and (d) HSP47 after treatment with 10% conditioned medium. Treatments were as follows: A: 10% cell-free medium or conditioned medium with no stimulation; C0, C1, C10, C100: 10% cell-free media or conditioned media containing IL-4 + IL-13 and PFD (0, 1, 10 or 100 µg/ml). Data are means ± SD of six experiments. All experiments were conducted in duplicate. ^#^*p*<0.01, compared with group A, and *p<0.01, compared with group C0.

PFD treatment had no effect on the levels of collagen type 1 (Fig. 7a) and HSP47 (Fig. 7c) in RLFs treated with 10% cell-free medium containing IL-4 + IL-13. However, the levels of these proteins significantly increased following treatment with 10% conditioned medium from NR8383 cells stimulated with IL-4 + IL-13. The levels of these proteins were significantly suppressed when RLFs were cultured with conditioned medium from NR8383 cells treated with PFD (10, 100 µg/ml; Fig. 7b and d).

## Discussion

This is the first study to show that PFD suppresses fibroblast proliferation through the inhibition of macrophage polarization towards the M2 phenotype, and not by the direct effect of the drug on the fibroblasts themselves. Although PFD did not affect M1 macrophage polarization, low concentrations of PFD (10 µg/ml) suppressed polarization towards the M2 phenotype, under our experimental conditions. Effective PFD doses observed in this study are in a similar range to the maximal plasma concentrations of this drug observed in humans when used at the recommended clinical dose.^(11)^

Macrophages can influence a variety of pathologies and are able acquire several phenotypes.^(27–29)^ Among the various macrophage phenotypes, the M2 phenotype has been shown to be associated with the secretion of pro-fibrotic factors and the promotion the fibroblast proliferation.^(19)^ M2 macrophage numbers have been reported to be elevated in lung tissue in a mouse model of pulmonary fibrosis.^(30)^ Therefore, the inhibitory effects of PFD observed in our study are of potential relevance for the suppression of lung fibrosis.

The increase in protein expression of M2 phenotype markers in NR8383 cells was suppressed following PFD treatment and was approximately 90–110% of the level observed for naïve cells (unstimulated macrophage) with a PFD concentration of 10 µg/ml, and approximately 75–90% with a PFD concentration of 100 µg/ml (Fig. 3). It has been suggested that PFD not only suppresses IL-4 + IL-13-dependent macrophage polarization towards the M2 phenotype, but also the inherent propensity for naïve cells to polarize towards this phenotype.^(31)^ Similarly, we have shown that PFD can suppress the increase in TGF-β1 levels induced by IL-4 + IL-13 (Fig. 4). TGF-β1 is secreted by M2 macrophages^(32)^ and has been shown to induce polarization from the M0 to the M2 phenotype by autocrine action.^(33)^ PFD may suppress both macrophage polarization towards the M2 phenotype and TGF-β1 levels secreted from M2 macrophages through its effects on paracrine and autocrine signaling.

Although it had been reported that the proliferation of fibroblast *in vitro* can be attenuated following treatment with PFD at concentrations of 300–1,000 µg/ml,^(12,13)^ in our experiments 1,000 µg/ml PFD showed toxicity towards fibroblasts (Fig. 1b). The addition of 10% cell-free medium containing 10 µg/ml PFD did not affect RLF proliferation (Fig. 5a and c). Although conditioned medium from NR8383 cells treated with IL-4 + IL-13 promoted RLF proliferation, the same conditioned medium from NR8383 cells that had also been treated with PFD (10 µg/ml) suppressed RLF proliferation significantly (Fig. 5d). These results suggest that low doses of PFD may be effective in suppressing the proliferation of fibroblasts by suppressing macrophage polarization towards the M2 phenotype. However, *Col1a1* and *HSP47* mRNA expression was suppressed by the addition of conditioned medium treated with the highest concentration of PFD (Fig. 6), whereas collagen type1 and HSP47 protein levels were significantly suppressed by the addition of conditioned medium treated with the lowest dose of PFD (10 µg/ml; Fig. 7b and d). HSP47 transiently associates with procollagen and is involved in collagen processing.^(26)^ Therefore, these results suggest that PFD post-transcriptionally suppressed the collagen triple helix by suppressing polarization to M2 phenotype macrophages. Furthermore, various bio-activators derived from NR8383 cells are affected by PFD, and can suppress the expression of fibrogenesis-related proteins in RLFs. In biological fibrosis environment, macrophages in closed to fibroblasts may release much higher concentrations of various bio-activators. These mechanisms should be examined in detail in the future.

Supplemental Fig. 1* showed that PFD treatment significantly decreased the hydroxyproline content and the levels of an M2 phenotypic marker in bleomycine (BLM)-treated rat lung tissue at concentrations of 10 mg/kg or more.

The mortality of acute exacerbations of IPF (AE-IPF) is a serious clinical problem. Kimura *et al.*^(34)^ established a BLM-induced mouse model for AE-IPF triggered by LPS. Because LPS promoted macrophage polarization towards the M1 phenotype (Fig. 2), it is possible that both M2 phenotype macrophages (induced by BLM) and M1 phenotype macrophages (induced by LPS) exist in AE-IPF lung. In our study, PFD did not suppress the polarization of M1 macrophages (Fig. 2), which release pro-inflammatory chemokines to amplify the inflammatory response.^(16)^ Clinically, it has been reported that PFD is not effective in AE-IPF patients with acute inflammation.^(35)^ Therefore, using PFD in combination with other drugs that suppress the activation of M1 macrophages or the inflammatory response^(36)^ may prove to be a more effective therapeutic strategy for AE-IPF.

In conclusion, this study has shown that clinically relevant concentrations of PFD can suppresses the proliferation and collagen levels of fibroblasts *in vitro* through the suppression of macrophage polarization towards the M2 phenotype. Further elucidation of the suppressive mechanisms by which PFD exerts its effects on macrophages and further study of the effects of PFD on macrophage polarization *in vivo* may lead to the development of more effective drugs for the treatment of fibrosis as well as other macrophage-related diseases.^(26–28)^

## Abbreviations

BLM: bleomycine
DMSO: dimethyl sulfoxide
FBS: fetal bovine serum
GAPDH: glyceraldehyde-3-phosphate dehydrogenase
HSP47: heat shock protein 47
IFN-γ: interferon-γ
IL-4: interleukin-4
IL-13: interleukin-13
iNOS: inducible nitric oxide synthase
IPF: idiopathic pulmonary fibrosis
IRP-1: iron regulatory protein-1
LPS: lipopolysaccharide
PFD: pirfenidone
PVDF: polyvinylidene difluoride
RLF: rat lung fibroblast
TGF-β1: transforming growth factor β1
TNF-α: tumor necrosis factor-α
WST-8: 2-(2-methoxy-4-nitrophenyl)-3-(4-nitrophenyl)-5-(2,4-disulfophenyl)-2H-tetrazolium, monosodium salt
YM-1: chitinase 3-like protein 3 + chitinase 3-like protein 4

## References

[1] Hutchinson J, Fogarty A, Hubbard R, McKeever T (2015). Global incidence and mortality of idiopathic pulmonary fibrosis: a systematic review. Eur Respir J.

[2] Richeldi L, du Bois RM, Raghu G (2014). Efficacy and safety of nintedanib in idiopathic pulmonary fibrosis. N Engl J Med.

[3] Hiroshima K, Iyoda A, Shibuya K (2002). Prognostic significance of neuroendocrine differentiation in adenocarcinoma of the lung. Ann Thorac Surg.

[4] Cottin V (2013). The role of pirfenidone in the treatment of idiopathic pulmonary fibrosis. Respir Res.

[5] Hasdemir PS, Ozkut M, Guvenal T (2017). Effect of pirfenidone on vascular proliferation, inflammation and fibrosis in an abdominal adhesion rat model. J Invest Surg.

[6] Hisatomi K, Mukae H, Sakamoto N (2012). Pirfenidone inhibits TGF-β1-induced over-expression of collagen type I and heat shock protein 47 in A549 cells. BMC Pulm Med.

[7] Schaefer CJ, Ruhrmund DW, Pan L, Seiwert SD, Kossen K (2011). Antifibrotic activities of pirfenidone in animal models. Eur Respir Rev.

[8] Iyer SN, Gurujeyalakshmi G, Giri SN (1999). Effects of pirfenidone on transforming growth factor-beta gene expression at the transcriptional level in bleomycin hamster model of lung fibrosis. J Pharmacol Exp Ther.

[9] Albera C, Costabel U, Fagan EA (2016). Efficacy of pirfenidone in patients with idiopathic pulmonary fibrosis with more preserved lung function. Eur Respir J.

[10] Noble PW, Albera C, Bradford WZ (2011). Pirfenidone in patients with idiopathic pulmonary fibrosis (CAPACITY): two randomised trials. Lancet.

[11] Lasky J (2004). Pirfenidone. IDrugs.

[12] Kim H, Choi YH, Park SJ (2010). Antifibrotic effect of Pirfenidone on orbital fibroblasts of patients with thyroid-associated ophthalmopathy by decreasing TIMP-1 and collagen levels. Invest Ophthalmol Vis Sci.

[13] Nakayama S, Mukae H, Sakamoto N (2008). Pirfenidone inhibits the expression of HSP47 in TGF-beta1-stimulated human lung fibroblasts. Life Sci.

[14] Lech M, Anders HJ (2013). Macrophages and fibrosis: How resident and infiltrating mononuclear phagocytes orchestrate all phases of tissue injury and repair. Biochim Biophys Acta.

[15] Martinez FO, Gordon S (2014). The M1 and M2 paradigm of macrophage activation: time for reassessment. F1000Prime Rep.

[16] Wynn TA, Barron L (2010). Macrophages: master regulators of inflammation and fibrosis. Semin Liver Dis.

[17] Ricardo SD, van Goor H, Eddy AA (2008). Macrophage diversity in renal injury and repair. J Clin Invest.

[18] Fadok VA, Bratton DL, Konowal A, Freed PW, Westcott JY, Henson PM (1998). Macrophages that have ingested apoptotic cells *in vitro* inhibit proinflammatory cytokine production through autocrine/paracrine mechanisms involving TGF-beta, PGE2, and PAF. J Clin Invest.

[19] Vernon MA, Mylonas KJ, Hughes J (2010). Macrophages and renal fibrosis. Semin Nephrol.

[20] Yamamoto-Oka H Mizuguchi S Toda M Carbon monoxide-releasing molecule, CORM-3, modulates alveolar macrophage M1/M2 phenotype *in vitro*. Inflammopharmacology. 2017. DOI: 10.1007.s10787-017-0371-y 10.1007/s10787-017-0371-y 28674739

[21] Helmke RJ, German VF, Mangos JA (1989). A continuous alveolar macrophage cell line: comparisons with freshly derived alveolar macrophages. In Vitro Cell Dev Biol.

[22] Chen JF, Ni HF, Pan MM (2013). Pirfenidone inhibits macrophage infiltration in 5/6 nephrectomized rats. Am J Physiol Renal Physiol.

[23] Geng Y, Zhang L, Fu B (2014). Mesenchymal stem cells ameliorate rhabdomyolysis-induced acute kidney injury via the activation of M2 macrophages. Stem Cell Res Ther.

[24] Corna G, Campana L, Pignatti E (2010). Polarization dictates iron handling by inflammatory and alternatively activated macrophages. Haematologica.

[25] Beljaars L, Schippers M, Reker-Smit C (2014). Hepatic localization of macrophage phenotypes during fibrogenesis and resolution of fibrosis in mice and humans. Front Immunol.

[26] Nagata K (2003). HSP47 as a collagen-specific molecular chaperone: function and expression in normal mouse development. Semin Cell Dev Biol.

[27] Qian L, Wang XY, Thapa S (2017). Macrophage migration inhibitory factor promoter polymorphisms (–794 CATT5–8): relationship with soluble MIF levels in coronary atherosclerotic disease subjects. BMC Cardiovasc Disord.

[28] García-Pavón S, Yamazaki-Nakashimada MA, Báez M, Borjas-Aguilar KL, Murata C (2017). Kawasaki disease complicated with macrophage activation syndrome: a systematic review. J Pediatr Hematol Oncol.

[29] Buscher K, Ehinger E, Gupta P (2017). Natural variation of macrophage activation as disease-relevant phenotype predictive of inflammation and cancer survival. Nat Commun.

[30] Gibbons MA, MacKinnon AC, Ramachandran P (2011). Ly6Chi monocytes direct alternatively activated profibrotic macrophage regulation of lung fibrosis. Am J Respir Crit Care Med.

[31] Staitieh BS, Egea EE, Fan X, Azih N, Neveu W, Guidot DM (2015). Activation of alveolar macrophages with interferon-γ promotes antioxidant defenses via the Nrf2-ARE pathway. J Clin Cell Immunol.

[32] Lugo-Villarino G, Vérollet C, Maridonneau-Parini I, Neyrolles O (2011). Macrophage polarization: convergence point targeted by *Mycobacterium tuberculosis* and HIV. Front Immunol.

[33] Arango Duque G, Descoteaux A (2014). Macrophage cytokines: involvement in immunity and infectious diseases. Front Immunol.

[34] Kimura T, Nojiri T, Hosoda H (2015). Exacerbation of bleomycin-induced injury by lipopolysaccharide in mice: establishment of a mouse model for acute exacerbation of interstitial lung diseases. Eur J Cardiothorac Surg.

[35] Iwata T, Yoshida S, Fujiwara T (2016). Effect of perioperative pirfenidone treatment in lung cancer patients with idiopathic pulmonary fibrosis. Ann Thorac Surg.

[36] Ninomiya K, Hayama K, Ishijima SA (2013). Suppression of inflammatory reactions by terpinen-4-ol, a main constituent of tea tree oil, in a murine model of oral candidiasis and its suppressive activity to cytokine production of macrophages *in vitro*. Biol Pharm Bull.

