# Supplementary material for: Pirfenidone suppresses polarization to M2 phenotype macrophages and the fibrogenic activity of rat lung fibroblasts
Source: J Clin Biochem Nutr. 2018 Apr 11;63(1):58–65. doi: 10.3164/jcbn.17-111 (PMC6064814; doi:10.3164/jcbn.17-111)
Supplement: Supplemental Fig. 1 [file jcbn17-111sf01.pdf]

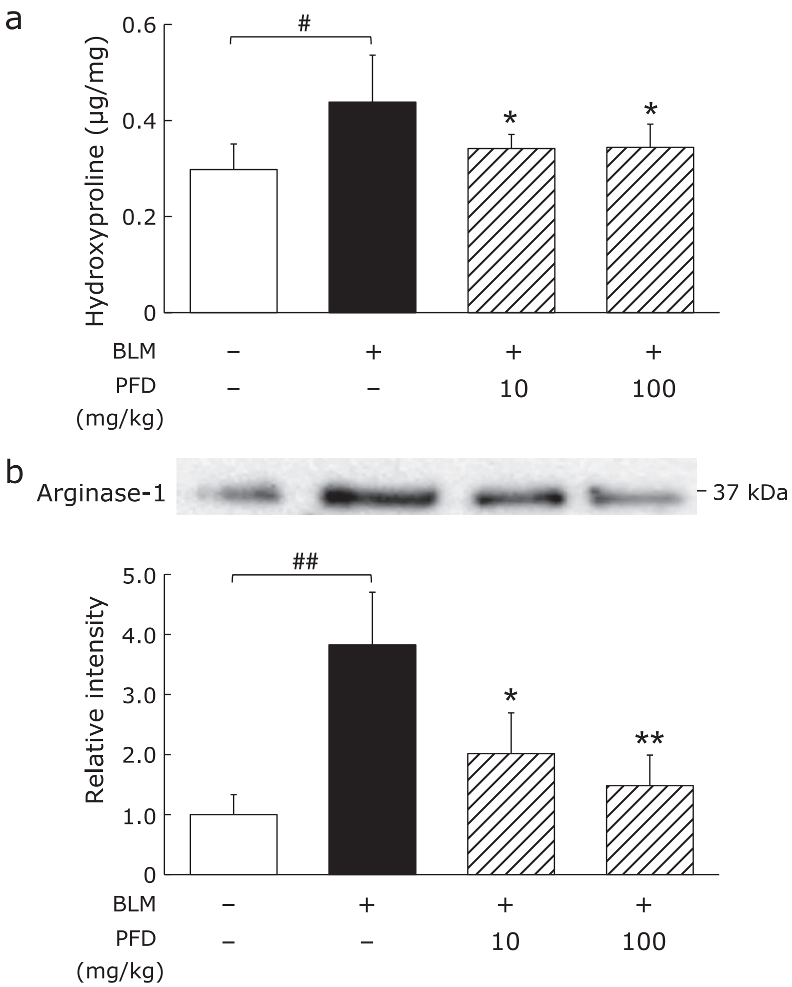

Supplemental Fig. 1. Effects of PFD on hydroxyproline contents and M2 phenotype macrophages in BLM-treated rat lung. Male Wistar rats (9 weeks old) were divided into four groups: saline + vehicle (sham), BLM (Nippon Kayaku, Tokyo, Japan) + vehicle, and BLM + PFD (10 or 100 mg/kg/day) dissolved in water containing 0.5% calboxymethyl cellulose (CMC). The rats were anesthetized with sevoflurane delivered in a box, and BLM (3 mg/kg) in 500 μl of saline or vehicle alone was administered via oropharyngeal aspiration (day 0) [Bale, *et al*. Indian J Pharmacol 2016; 48: 643–648]. Treatments (vehicle or PFD) were started 1 day before the BLM instillation. Animals were sacrificed at day 14. The collection of specimens from rats was performed as described previously [Mizuguchi, *et al*. Biofactor 2006; 26: 81–92]. (a) Hydroxyproline content was measured as described previously [Mizuguchi, *et al*. Biofactor 2006; 26: 81–92]. (b) The levels of the M2 marker, arginase-1 (anti-arginase-1 antibody, cat. #ab92274, Abcam). Data are means ± SD of at least five experiments. $^{\#}p<0.05$ and $^{\#\#}p<0.01$, compared with the sham group. $^{*}p<0.05$ and $^{**}p<0.01$, compared with the BLM treated group.
